# Electrochemical oxidation of ferricyanide

**DOI:** 10.1038/s41598-021-02355-3

**Published:** 2021-11-29

**Authors:** Mun Hon Cheah, Petko Chernev

**Affiliations:** Molecular Biomimetics, Department of Chemistry – Ångström Laboratory, Uppsala University, Box 523, 75120 Uppsala, Sweden

**Keywords:** Chemistry, Coordination chemistry, Electrochemistry, Inorganic chemistry

## Abstract

We report the electrochemical oxidation of ferricyanide, [Fe^III^(CN)_6_]^3−^ and characterised the oxidation product by in-situ FTIR and XAS spectroelectrochemistry methods. Oxidation of [Fe^III^(CN)_6_]^3−^ is proposed to proceed via a tentative Fe(IV) intermediate that undergoes reduction elimination to give cis-[Fe^III^(CN)_4_(CH_3_CN)_2_]^1−^ as stable product in acetonitrile. Speciation of the oxidation product by DFT calculations is underpinned by good agreement to experimental data.

## Introduction

Hexacyano ferrate(III), [Fe^III^(CN)_6_]^3−^, or ferricyanide is a stable, low spin Fe(III) complex that is well-known for its reversible reduction to hexacyano ferrate(II), [Fe^II^(CN)_6_]^4−^ or ferrocyanide and for its reaction with aqueous Fe(II) to form Prussian Blue complexes. Both [Fe^III^(CN)_6_]^3−^ and [Fe^II^(CN)_6_]^4−^ are widely used in fundamental studies such as study of metal ligand bonding by XAS^1,2^, benchmarks for computational modelling^3–5^ or in practical applications such as redox buffer in biological studies^6^ or thermogalvanic cells^7^. From redox chemistry perspective, the most wide spread use of ferricyanide or ferricyanide is as single electron acceptor or donor respectively due to the well-known [Fe^III/II^(CN)_6_]^3−/4−^ reversible couple. The reduction potential of [Fe^III^(CN)_6_]^3−^ is highly tunable and is reported to be dependent on solvent^8–12^, cations^7,13^ or formation of borane adducts^11^ leading to suggestion that this redox couple can be used as a versatile and tunable redox mediator^11,12^. Indeed, when transferred from water solvent into acetonitrile, the reduction potential of [Fe^III^(CN)_6_]^3−^ is shifted more negative by almost 1 V^8–12^. Despite this interesting aspect, all redox chemistry of [Fe^III^(CN)_6_]^3−^ reported to date is exclusively focused on it reversible reduction to [Fe^II^(CN)_6_]^4−^. Here we report, to the best of our knowledge, the first electrochemical oxidation of [Fe^III^(CN)_6_]^3−^ in non-aqueous solvent, thus extending our knowledge beyond the well-known [Fe^III/II^(CN)_6_]^3−/4−^ reversible couple. We characterized the oxidation product of [Fe^III^(CN)_6_]^3−^ by a combination of in-situ FTIR and XAS spectroelectrochemistry and use DFT calculations to aid interpretation of the spectroscopic data.

## Results and discussion

**Figure 1:**
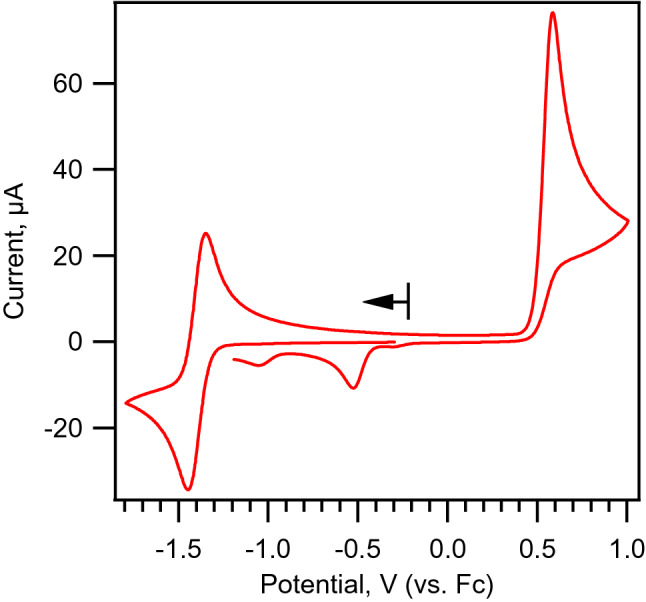
Cyclic voltammogram of 2 mM [(C_4_H_9_)_4_N]_3_[Fe^III^(CN)_6_] in anhydrous acetonitrile. Scan rate: 100 mV/s. 3 mm diameter GC working electrode.

**Figure 2:**
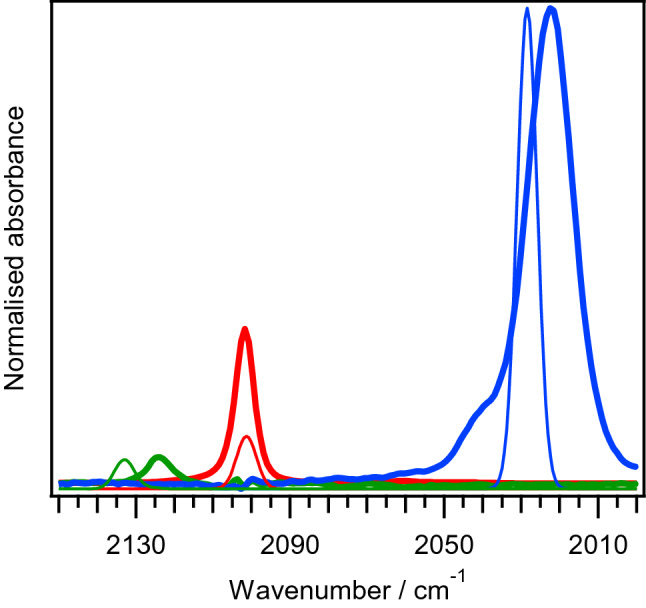
FTIR spectra of [Fe^II^(CN)_6_]^4−^ (blue), [Fe^III^(CN)_6_]^3−^ (red), **ox-ferri** (green) obtained from IR-SEC (thick lines). Thin lines: simulated IR spectra of [Fe^II^(CN)_6_]^4−^ (blue), [Fe^III^(CN)_6_]^3−^ (red), cis-[Fe^III^(CN)_4_(CH_3_CN)_2_]^1−^ (green) from DFT calculations.

Cyclic voltammetry of a dry acetonitrile solution of [(C_4_H_9_)_4_N]_3_[Fe^III^(CN)_6_] is depicted in Fig. 1. In the cathodic direction, a reversible redox couple centered at − 1.397 V (vs. Fc^+^/Fc couple) is observed. This redox couple has been assigned as the reversible reduction of [Fe^III^(CN)_6_]^3−^ to [Fe^II^(CN)_6_]^4−^ based on previous literature reports^8–12^. We have independently verified this assignment by FTIR spectroelectrochemistry (IR-SEC). Reduction of [(C_4_H_9_)_4_N]_3_[Fe^III^(CN)_6_] in anhydrous acetonitrile result in depletion of intensity of a band at 2102 cm^−1^ and concomitant intensity increase of a major band at 2022 cm^−1^ (Fig. 2). Upon re-oxidation, we observed quantitative recovery of the starting material, consistent with the observed reversibility in cyclic voltammetry experiments. The observed changes in the νCN frequencies and band intensities are consistent with those observed for the reversible oxidation of K_4_[Fe^II^(CN)_6_] in water, where bands at 2038 and 2115 cm^−1^ are attributed to [Fe^II^(CN)_6_]^4−^ and [Fe^III^(CN)_6_]^3−^ respectively (Fig. S3, ESI). Overall, we observed the same trend where reduction of [Fe^III^(CN)_6_]^3−^ to [Fe^II^(CN)_6_]^4−^ resulted in lowering the νCN frequency by 80 cm^−1^ in acetonitrile and 77 cm^−1^ in water. We attribute the offset in νCN frequencies of the acetonitrile and water dataset of approximately 13 cm^−1^ to solvent and counter cation effects^14^. Additionally, there is a minor shoulder feature at 2042 cm^−1^ which was attributed to adsorption of [Fe^II^(CN)_6_]^4−^ to electrode surface in a previous SEC study^15^.

On the anodic scan direction in the cyclic voltammogram, an irreversible oxidation wave is observed at 0.586 V. Upon reversing the scan direction from anodic to cathodic, three additional reduction peaks at − 0.307 V, − 0.524 V and − 1.050 V are observed. These additional peaks are attributed to reduction of daughter products of the irreversible oxidation at 0.586 V as they are only observable in the presence of this irreversible oxidation peak. Comparison of the peak currents between the reversible [Fe^III^/Fe^II^(CN)_6_]^3−/4−^ couple and irreversible oxidation peak at 0.586 V reveals a ratio of 1:2.2, suggesting an apparent, overall two electron oxidation process associated with the irreversible oxidation peak. We conducted IR-SEC study on the oxidation process associated with the irreversible oxidation peak at 0.586 V. Oxidation of [(C_4_H_9_)_4_N]_3_ [Fe^III^(CN)_6_] result in depletion in intensity of the [Fe^III^(CN)_6_]^3−^ νCN band at 2102 cm^−1^ and formation of a new species (here on in referred to as **ox-ferri**) with single νCN band at 2124 cm^−1^ (Fig. 2). The increase in νCN frequency by 22 cm^−1^ is smaller than the expect shift associated with a single electron oxidation of approximately 70–80 cm^−1^, as evident by the observed shift in νCN frequency of the [Fe^III^/Fe^II^(CN)_6_]^3−/4−^ couple.

**Figure 3:**
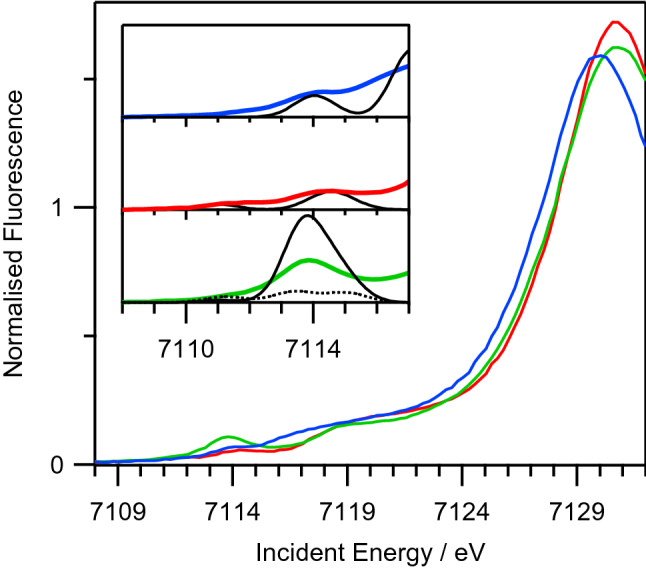
Fe K-edge XAS spectra of [Fe^II^(CN)_6_]^4−^ (blue), [Fe^III^(CN)_6_]^3−^ (red) and **ox-ferri** (green). Inset: Fe K-edge 1*s* → 3*d* pre-edge region, experimental data are plotted with thick lines. Simulated spectra from TD-DFT calculations plotted in thin black lines. For **ox-ferri**, simulated spectrum of the cis-[Fe^III^(CN)_4_(CH_3_CN)_2_]^1−^ isomer with C_2v_ symmetry plotted with solid thin line and the trans-isomer with D_4h_ symmetry plotted with thin dashed line.

Oxidation of [Fe^III^(CN)_6_]^3−^ is expected to be metal centered as the HOMO is the metal based t_2g_ orbital. Metal centered, one or two-electron oxidation of [Fe^III^(CN)_6_]^3−^ will result in formation of a Fe(IV) or Fe(V) species, which is contradicted by the relatively small increase in νCN frequency of 22 cm^−1^ observed in IR-SEC experiment above. To characterise the Fe oxidation state of **ox-ferri** in acetonitrile, we conducted Fe K-edge XAS-SEC studies on **ox-ferri**, as well as the reversible [Fe^III^/Fe^II^(CN)_6_]^3−/4−^ couple as reference samples. The Fe K-edge position of **ox-ferri** is at 7125.9 eV, close to that observed for [Fe^III^(CN)_6_]^3−^ (7126.1 eV) while the edge for [Fe^II^(CN)_6_]^4−^ is at 7125.1 eV (Fig. 3). The Fe K-edge position of **ox-ferri**, which is slightly lower to that of [Fe^III^(CN)_6_]^3−^ is inconsistent with the presence of either Fe(IV) or Fe(V) oxidation state. Instead, it is likely that **ox-ferri** is in the Fe(III) oxidation state.

Based on the observations above, we propose that the irreversible oxidation peak observed at 0.586 V is the result of an ECE type reaction:

$${\left[{\mathrm{Fe}}^{\mathrm{III}}{\left(\mathrm{CN}\right)}_{6}\right]}^{3-}\rightleftharpoons {\left[{\mathrm{Fe}}^{\mathrm{IV}}{\left(\mathrm{CN}\right)}_{6}\right]}^{2-} + {\mathrm{ e}}^{-}$$

$${\left[{\mathrm{Fe}}^{\mathrm{IV}}{\left(\mathrm{CN}\right)}_{6}\right]}^{2-}\rightleftharpoons {\left[{\mathrm{Fe}}^{\mathrm{II}}{\left(\mathrm{CN}\right)}_{4}\right]}^{2-}+{\left(\mathrm{CN}\right)}_{2}$$

$${\left[{\mathrm{Fe}}^{\mathrm{II}}{\left(\mathrm{CN}\right)}_{4}\right]}^{2-}\rightleftharpoons {\left[{\mathrm{Fe}}^{\mathrm{III}}{\left(\mathrm{CN}\right)}_{4}\right]}^{1- } + {\mathrm{ e}}^{-}$$

The first electron transfer step (E) is the metal centered oxidation of [Fe^III^(CN)_6_]^3−^ to a [Fe^IV^(CN)_6_]^2−^ intermediate. This intermediate then undergoes a chemical step (C) which we propose as reductive elimination reaction to release cyanogen, (CN)_2_, to form a [Fe^II^(CN)_4_]^2−^ intermediate. The [Fe^II^(CN)_4_]^2−^ intermediate has an oxidation potential that is lower compared to the first E step and is immediately oxidised at the electrode to give the final product [Fe^III^(CN)_4_]^1−^. This second E step with lower oxidation potential is consistent with the presence of daughter products observed at lower reduction potentials during the reverse scan. This proposed ECE reaction can account for the appearance of an apparent single, two-electron oxidation peak that is irreversible in the voltammogram while the final product is a Fe(III) species that is consistent with the observed Fe K-edge position of **ox-ferri**. Lending further support to our hypothesis of a reductive elimination reaction forming a [Fe^III^(CN)_4_]^1−^ species, there is a more intense pre-edge peak at 7113.9 eV for **ox-ferri** compared to the pre-edge peaks observed for [Fe^III^(CN)_6_]^3−^ and [Fe^II^(CN)_6_]^4−^ at the Fe K-edge 1*s* → 3*d* region (Fig. 3). This increase in pre-edge intensity is likely reflecting a change from centrosymmetric environments for [Fe^III^(CN)_6_]^3−^ and [Fe^II^(CN)_6_]^4−^ to non-centrosymmetric environment for **ox-ferri**^1^. This observation appears consistent with our proposal of a [Fe^III^(CN)_4_]^1−^ species, assuming such species has T_d_ symmetry.

**Figure 4:**
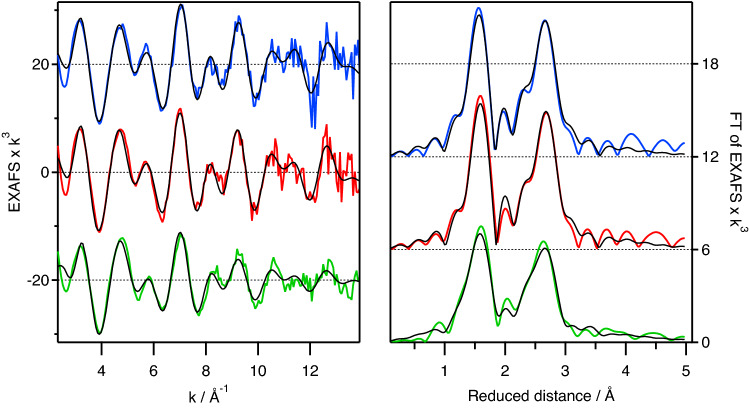
EXAFS (k^3^-weighted) of [Fe^II^(CN)_6_]^4−^ (blue), [Fe^III^(CN)_6_]^3−^ (red), **ox-ferri** (green). Data offset for clarity. Simulated spectra for DFT-optimized structures plotted in black lines, with the **ox-ferri** simulation based on the cis-[Fe^III^(CN)_4_(CH_3_CN)_2_]^1−^ isomer with C_2v_ symmetry. Fit parameters are given in Table S3.

However, fitting of the EXAFS of **ox-ferri** using structural models of [Fe^III^(CN)_4_]^1−^ with either T_d_ or D_4h_ symmetry resulted in unsatisfactory fits (Fig. S4, ESI). A plausible explanation of the poor EXAFS fits maybe due to additional ligand binding to the coordinative unsaturated Fe center of [Fe^III^(CN)_4_]^1−^ to form either five or six coordinate species. The additional ligands are likely to be acetonitrile solvent or polymerisation of [Fe^III^(CN)_4_]^1−^ to form Prussian Blue like networks. Since the EXAFS of **ox-ferri** does not show any Fe–Fe interaction at 5 Å (Fig. 4) that is indicative of formation of Prussian Blue^16,17^, this suggests that **ox-ferri** is most likely a solvent coordinated [Fe^III^(CN)_4_(CH_3_CN)_x_]^1−^, x = 1 or 2 species. Similar tetracyano [Fe^II/III^(CN)_4_(L)_2_]^2−/1−^ species, where L are CO or solvent molecules such as DMSO and pyridine were previously reported with no evidence of formation of Prussian Blue like networks^18,19^.

To facilitate speciation of **ox-ferri**, we optimized a series of structures with [Fe^III^(CN)_4_(CH_3_CN)_x_]^1−^, x = 0, 1 or 2, using DFT calculations and compared the calculated structural and spectroscopic parameters to available experimental data. The validity of this approach is underpinned by the good agreement between calculated and experimental data using [Fe^III^(CN)_6_]^3−^ and [Fe^II^(CN)_6_]^4−^ as reference dataset (ESI). The optimized structures of [Fe^III^(CN)_4_(CH_3_CN)_x_]^1−^, x = 0, 1 or 2, species are presented in Fig. S5, ESI.

First, comparison of the calculated relative energies suggest a stabilization effect due to coordination of acetonitrile solvents to [Fe^III^(CN)_4_]^1−^ species with binding of a single acetonitrile stabilizing all [Fe^III^(CN)_4_(CH_3_CN)_1_]^1−^ complexes by approximately 20 kcal/mol on average. Binding of a second acetonitrile further stabilizes all [Fe^III^(CN)_4_(CH_3_CN)_2_]^1−^ complexes by approximately 13 kcal/mol on average. These values are within reported range of binding energies of acetonitrile or the closely related CO to metal centers^20–22^ and are consistent with our proposal of **ox-ferri** being a solvent coordinated complex. For the lowest energy [Fe^III^(CN)_4_(CH_3_CN)_2_]^1−^ complexes, two possible isomers (cis-isomer with C_2v_ symmetry and trans-isomer with D_4h_ symmetry) exist with the cis-isomer being 2.67 kcal/mol lower in energy.

We compare the simulated metal cyanide νCN vibration frequencies and intensities of the various calculated structures of [Fe^III^(CN)_4_(CH_3_CN)_x_]^1−^, x = 0, 1 or 2, with experimental values of **ox-ferri** (Fig. 2 and Fig. S6, ESI). The simulated νCN vibration spectra of all isomers of [Fe^III^(CN)_4_(CH_3_CN)_1_]^1−^ are a poor match to experimental values, with some isomers exhibiting two νCN bands instead. The calculated νCN vibration frequencies of all isomers of [Fe^III^(CN)_4_]^1−^ and [Fe^III^(CN)_4_(CH_3_CN)_2_]^1−^ can be considered as satisfactory match to the experimental value, with the calculated intensities for isomers of [Fe^III^(CN)_4_]^1−^ significantly overestimated compared to those of [Fe^III^(CN)_4_(CH_3_CN)_2_]^1−^. Based on these observations, we can rule out all [Fe^III^(CN)_4_(CH_3_CN)_1_]^1−^ species as candidate for **ox-ferri**. However, it is not possible to distinguish between structural isomers of [Fe^III^(CN)_4_]^1−^ and [Fe^III^(CN)_4_(CH_3_CN)_2_]^1−^ as potential candidate for **ox-ferri** based solely on comparison of νCN vibration spectra. We note that there should be in principle four active νCN vibration modes for the cis-isomer of [Fe^III^(CN)_4_(CH_3_CN)_2_]^1−^ based on vibrational mode analysis and IR selection rules^23^. DFT calculation of the cis-isomer of [Fe^III^(CN)_4_(CH_3_CN)_2_]^1−^, indeed shows 4 active νCN vibration modes with wavenumbers close to each other. However the intensity of the b_1_ vibration mode is significantly higher relative to the remaining 2a_1_, b_2_ vibration modes, leading to the appearance of a single νCN vibration band in the simulated spectrum. Similar experimental observation where only a single observable νCN band for the closely related cis-[Fe^III^(CN)_4_(pyridine)_2_]^1−^ complex (with C_2v_ symmetry) has been reported^18^.

Next we compare structural parameters of calculated structures for [Fe^III^(CN)_4_(CH_3_CN)_x_]^1−^, x = 0, 1 or 2 to experimental EXAFS data. The approach adopted in this study is to compare simulated EXAFS spectra, based on DFT optimized structures, to the experimental EXAFS spectrum of **ox-ferri**. As mentioned above, all structures of [Fe^III^(CN)_4_]^1−^ give a poor match between simulated and experimental EXAFS (Fig. S4, ESI) and can be excluded as candidates for **ox-ferri**. EXAFS simulations of all isomers of [Fe^III^(CN)_4_(CH_3_CN)_1_]^1−^ and [Fe^III^(CN)_4_(CH_3_CN)_2_]^1−^ (Fig. S4, ESI) gives satisfactory match to the experimental spectrum, with simulations using isomers of [Fe^III^(CN)_4_(CH_3_CN)_2_]^1−^ resulting in slightly lower RMSD. Taking into account the comparison of the νCN vibration frequencies above, we can additionally rule out all [Fe^III^(CN)_4_(CH_3_CN)_1_]^1−^ species as candidates for **ox-ferri**, thus leaving [Fe^III^(CN)_4_(CH_3_CN)_2_]^1−^ species as candidates for **ox-ferri**. However, the simulated EXAFS for both isomers of [Fe^III^(CN)_4_(CH_3_CN)_2_]^1−^ are almost indistinguishable and cannot provide unambiguous assignment towards identity of **ox-ferri**.

Finally we compare the TD-DFT calculated peak position and intensities of Fe 1*s* → 3*d* transitions for all isomers of [Fe^III^(CN)_4_(CH_3_CN)_x_]^1−^, x = 0, 1 or 2 and compare them to the experimental peaks in the pre-edge region of Fe K-edge XANES of **ox-ferri**. Of all calculated structures, only the cis-isomer of [Fe^III^(CN)_4_(CH_3_CN)_2_]^1−^ gives a good match between observed and calculated peak position and intensity in the pre-edge region (Fig. 3). The calculated peak intensity at 7113.9 eV is predominantly due to electric dipole allowed transition and is approximately two times larger compared to experiment. This is consistent with a previous report where dipole allowed transition intensities are typically overestimated in TD-DFT calculations^4^. Furthermore, the good match between observed and calculated pre-edge peaks for the cis-isomer of [Fe^III^(CN)_4_(CH_3_CN)_2_]^1−^ is fully consistent with the observed increase in pre-edge intensity going from [Fe^III^(CN)_4_]^3−^ to **ox-ferri** as mentioned above. The cis-isomer of [Fe^III^(CN)_4_(CH_3_CN)_2_]^1−^ has non-centrosymmetric Fe center due to its C_2v_ symmetry whilst the trans-isomer of [Fe^III^(CN)_4_(CH_3_CN)_2_]^1−^ retains the centrosymmetric Fe center due to its D_4h_ symmetry. Taking into account the computed relative energy difference as well as the comparison between computed and experimental structural and spectroscopic parameters (νCN vibration frequency, EXAFS and XANES pre-edge peaks), we conclude that the most plausible candidate of **ox-ferri** is the cis-isomer of [Fe^III^(CN)_4_(CH_3_CN)_2_]^1−^.

## Conclusions

In conclusion, we report electrochemical oxidation reaction of [Fe^III^(CN)_6_]^3−^ in anhydrous acetonitrile and characterised the oxidation product, **ox-ferri**, by FTIR and XAS spectroelectrochemistry. We propose an ECE oxidation mechanism of [Fe^III^(CN)_6_]^3−^, implicating a Fe(IV) intermediate followed by reductive elimination of two cyanide ligands to form a [Fe^III^(CN)_4_(CH_3_CN)_2_]^1−^ species. The speciation of **ox-ferri** is aided by DFT calculations where we show that the calculated structural and spectroscopic parameters of the cis-[Fe^III^(CN)_4_(CH_3_CN)_2_]^1−^ isomer (C_2v_ symmetry) gives the best match to available experimental data. This study extends the redox chemistry of [Fe^III^(CN)_6_]^3−^ beyond the conventional reversible [Fe^III/II^(CN)_6_]^3−/4−^ couple, this in turn suggests new redox reactions may exist in closely related cyanometallates such as Prussian Blue and its analogues.

## Methods

### Synthesis of [(C_4_H_9_)_4_N]_3_[Fe^III^(CN)_6_]

[(C_4_H_9_)_4_N]_3_[Fe^III^(CN)_6_] was prepared according to literature procedure^14^.

### Cyclic voltammetry

Cyclic voltammetry was carried using an Autolab PGSTAT302N potentiostat with Nova 2.1 software. The 3-electrode cell consists of a 3 mm diameter glassy carbon working electrode (ALS Japan), a custom Ag/Ag^+^ pseudo reference electrode isolated from sample solution with a frit and a graphite rod counter electrode. The working electrode is polished with 0.05 micron alumina slurry in distilled water, sonicated in distilled water and dried by rinsing with dry acetonitrile immediately before use. A 7 mL solution of 2 mM [(C_4_H_9_)_4_N]_3_[Fe^III^(CN)_6_] with 200 mM tetrabutylammonium hexafluorophosphate solution in dry acetonitrile is purged with N_2_ gas that is bubbled through dry acetonitrile before use. The sample solution is kept under N_2_ atmosphere blanket throughout the experiment by gentle introduction of N_2_ into the headspace of the voltammetry cell.

### FTIR spectroelectrochemistry

FTIR spectroelectrochemistry (IR-SEC) was carried out using a custom-built spectroelectrochemical setup described previously^24^. The working electrode is a 3 mm diameter glassy carbon electrode, Ag wire pseudo reference and Pt counter electrode. The SEC cell was filled with sample solution inside a dry glovebox (Vacuum Atmospheres Company). The SEC cell was connected to either (i) a PAR model 362 potentiostat with a PowerLab 4/20 interface and IR spectra were collected on a Biorad FTS 175C FTIR spectrometer with Ge/KBr beamsplitter and MCT detector or (ii) Autolab PGSTAT302N potentiostat and Bruker Vertex 70v FTIR spectrometer equipped with Ge/KBr beamsplitter and MCT detector.

### XAS spectroelectrochemistry

XAS spectroelectrochemistry (XAS-SEC) was carried out using a purpose-built XAS-SEC cell constructed from PEEK and PTFE (Fig. S1). The working electrode compartment is made from PTFE and sandwiched between a PEEK faceplate and counter electrode compartment made from PEEK. The working electrode is a 10 × 10 × 3 mm reticulated vitreous carbon (RVC) foam block (Good Fellow Cambridge Ltd) housed in the PTFE block. A ‘leak free’ Ag/AgCl reference electrode (eDAQ) is inserted into the working electrode compartment, which is separated from the counter electrode compartment by a 0.3 mm thick filter paper. The counter compartment houses a 10 × 10 × 10 mm RVC foam block and is filled with dry acetonitrile solution of 200 mM tetrabutylammonium hexafluorphosphate. A 12 μm thick Kapton foil is sandwiched between the PEEK front faceplate and the PTFE working compartment to form the X-ray window. Sample solution (5 mM [(C_4_H_9_)_4_N]_3_[Fe^III^(CN)_6_] with 200 mM tetrabutylammonium hexafluorophosphate solution in dry acetonitrile) is introduced to the working compartment via a syringe pump and 1/16″ diameter PTFE tubing at a flow rate of 10 μL/min.

XAS spectra were collected at the Balder Beamline at MAX IV laboratory, Lund, Sweden. The incoming X-ray energy was scanned over the iron K-edge via a Si111 double crystal monochromator. The X-ray beam was defocused to a size of 0.5 mm × 3 mm to avoid radiation damage. The X-ray absorption was measured in fluorescence mode using an energy dispersive 7-element Si-drift detector with region of interest set at the iron Kα fluorescence. All measurements were done at room temperature. A 10 μm Fe foil (Goodfellow Cambridge Ltd) served as calibration standard and was measured several times per day to ensure a stable energy axis. Energy calibration was done by assigning the position of the maximum of the first derivative of the Fe foil absorption to 7112 eV.

The EXAFS spectra were simulated using the FEFF 9.0 software, with the following parameters: DEBYE 300 800, NLEG 6, CRITERIA 12 5, RPATH 4.6, SCF 7.0 1 30 0.05. Fitting was done with in-house software (SimXLite). EXAFS was weighted by k^3^ and fitted in k-range 2.3–14 Å^−1^. Coordination numbers were kept fixed to the values in the structural models. Debye–Waller parameters were fitted and had one value for first-shell-only paths and another value for the rest of the paths. To determine which DFT functional gives best results for this type of compounds, the [Fe^II^(CN)_6_]^4−^ and [Fe^III^(CN)_6_]^3−^ spectra were first fitted allowing the Fe-to-ligand distances to change (parameter values in Table S2, fitted spectra in Fig. S9). The distances determined in this way were then compared to distances obtained by DFT geometry optimization with different functionals (Table S1). The [Fe^II^(CN)_6_]^4−^ and [Fe^III^(CN)_6_]^3−^ spectra were then fitted again using the DFT-optimized structures, with no fitting of Fe-to-ligand distances, to ensure the validity of this approach and to determine an optimal value for the amplitude reduction factor S_0_^2^ (parameter values in Table S3, fitted spectra in Fig. 4 in the main text). Finally, for [Fe^III^(CN)_4_(CH_3_CN)_x_]^1−^, x = 0, 1 or 2, species, the EXAFS was simulated using geometry optimized structures from DFT calculations, with no fitting of Fe to ligand distances (parameter values in Table S3, fitted spectra in Fig. S4).

### DFT calculations

DFT calculation were performed on the Tetralith cluster, National Supercomputer Centre using the ORCA 4.2.1 software package^25^. All geometry optimization and numerical frequency calculations were performed using the TPSS functional^26^ and minimally augmented ma-def2-TZVP(-f) basis set^27^, with the f polarization removed from main group elements. Solvation effects were included using the SMD solvation model^28^ with parameters for acetonitrile. The resolution of identity approximation (RI-J)^29^ and AutoAux auxiliary basis set^30^ was used to reduce computational time. All geometry-optimized structures were confirmed to be at local minima with numerical frequency calculations. TD-DFT calculations were performed using TPSS/ma-def2-TZVP(-f) optimized structures with the ZORA approximation^31^, the corresponding ma-ZORA-def2-TZVP(-f) basis set^32^, B3LYP hybrid functional^33^ and SMD solvation model.

The selection of the TPSS functional for geometry optimization and numerical frequency calculations in this study is based on the good match between experimental and calculated structural parameters and νCN vibration frequencies for [Fe^II^(CN)_6_]^4−^ and [Fe^III^(CN)_6_]^3−^ (Fig. S8 and Table S1).

The simulated νCN bands are obtained by broadening the calculated νCN vibration frequencies and intensities with a Gaussian function with FWHM of 3 cm^−1^ with no offset in calculated vibration frequencies. The intensity of all simulated spectra are scaled based on a factor determined by normalising the simulated and experimental intensities of [Fe^II^(CN)_6_]^4−^. For the simulated spectra for Fe 1*s* → 3*d* transitions, all calculated peaks are broaden by 1.5 eV and peak positions are offset by + 25.5 eV. The peak intensities are scaled by a factor determined by normalizing the calculated peak intensity of the 1*s* → 3*d* (e_g_) transition to the corresponding experimentally observed peak at 7114.3 eV for [Fe^III^(CN)_6_]^3−^.

## Supplementary Information

Supplementary Information.

## Data Availability

The datasets generated during and/or analysed during the current study are available from the corresponding author on reasonable request.
