# Supplementary material for: Electrochemical oxidation of ferricyanide
Source: Sci Rep. 2021 Nov 29;11:23058. doi: 10.1038/s41598-021-02355-3 (PMC8629995; doi:10.1038/s41598-021-02355-3)
Supplement: Supplementary file 1 — Supplementary Information. [file 41598_2021_2355_MOESM1_ESM.pdf]

Electrochemical oxidation of ferricyanide

Mun Hon Cheah*[a] and Petko Chernev[a]

[a.] Molecular Biomimetics, Department of Chemistry – Ångström Laboratory, Uppsala University, 75120 Uppsala, Sweden

* Corresponding author:

Mun Hon Cheah, Molecular Biomimetics, Department of Chemistry – Ångström Laboratory, Uppsala University, Box 523, SE-75120 Uppsala, Sweden; phone: +46(73)8030682, email: michael.cheah@kemi.uu.se

Electronic supplementary information

Table of contents

Fig. S1 Schematic of in-situ XAS spectroelectrochemistry cell.

Fig. Cyclic voltammogram of $[(C_4H_9)_4N]_3[Fe^{III}(CN)_6]$ and background cyclic voltammogram

Fig. S3 IR-SEC difference spectra of (A) oxidation of $K_4[Fe^{II}(CN)_6]$ to $K_3[Fe^{III}(CN)_6]$ in water solvent. (B) Reduction of $[(C_4H_9)_4N]_3[Fe^{III}(CN)_6]$ to $[(C_4H_9)_4N]_4[Fe^{II}(CN)_6]$ in dry acetonitrile. (C) Oxidation of $[(C_4H_9)_4N]_3[Fe^{III}(CN)_6]$ to **ox-ferri** in dry acetonitrile.

Fig. S4 Experimental EXAFS of **ox-ferri** (green lines) and simulations of EXAFS using DFT optimized structures of $[Fe^{III}(CN)_4(MeCN)_x]^{1-}$, x = 1 or 2 species (black lines).

Fig. S5 DFT optimized structures and relative Gibbs free energy of $[Fe^{III}(CN)_4(MeCN)_x]^{1-}$, x = 0, 1 or 2.

Fig. S6 Top panel, experimental IR spectra obtained from IR-SEC (thick lines) and simulated IR spectra of $[Fe^{II}(CN)_6]^{4-}$ (blue), $[Fe^{III}(CN)_6]^{3-}$ (red), cis-$[Fe^{III}(CN)_4(CH_3CN)_2]^{1-}$ (green) from DFT calculations (thin lines). Bottom panel, simulated IR spectra of various geometry optimized $[Fe^{III}(CN)_4(MeCN)_x]^{1-}$, x = 1 or 2 species (thin lines).

Fig. S7 Fe K-edge 1s → 3d pre-edge region for $[Fe^{II}(CN)_6]^{4-}$ (blue line), $[Fe^{III}(CN)_6]^{3-}$ (red line) and **ox-ferri** (green line). Simulated spectra plotted in black lines.

Fig. S8 Comparison between calculated and experimental νCN vibration frequencies for $[Fe^{II}(CN)_6]^{4-}$ and $[Fe^{III}(CN)_6]^{3-}$ with different DFT functionals.

Fig. S9 Experimental and simulated EXAFS (k^3-weighted) of $[Fe^{II}(CN)_6]^{4-}$ (blue), $[Fe^{III}(CN)_6]^{3-}$ (red).

Table S1 Comparison between Fe-C and Fe-N distance obtained from geometry optimized structures of $[Fe^{II}(CN)_6]^{4-}$ and $[Fe^{III}(CN)_6]^{3-}$ using various DFT functionals to distance obtained from EXAFS fitting.

Table S2 EXAFS fit parameters for $[Fe^{II}(CN)_6]^{4-}$ and $[Fe^{III}(CN)_6]^{3-}$ used to determine Fe-C and Fe-N distances in the two reference structures.

Table S3 Debye-Waller parameters (σ, in Å) and goodness-of-fit value (R_f, in %) obtained from EXAFS fitting of DFT-optimized models of $[Fe^{II}(CN)_6]^{4-}$, $[Fe^{III}(CN)_6]^{3-}$ and **ox-ferri**.

xyz coordinates for DFT optimised $[Fe^{II}(CN)_6]^{4-}$, $[Fe^{III}(CN)_6]^{3-}$, cis-$[Fe^{III}(CN)_4(CH_3CN)_2]^{1-}$

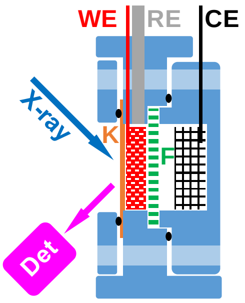

Fig. S1 Schematic of in-situ XAS spectroelectrochemistry cell. WE: Working electrode, RE: reference electrode, CE: counter electrode, K: Kapton window, F: filter paper, Det: X-ray detector

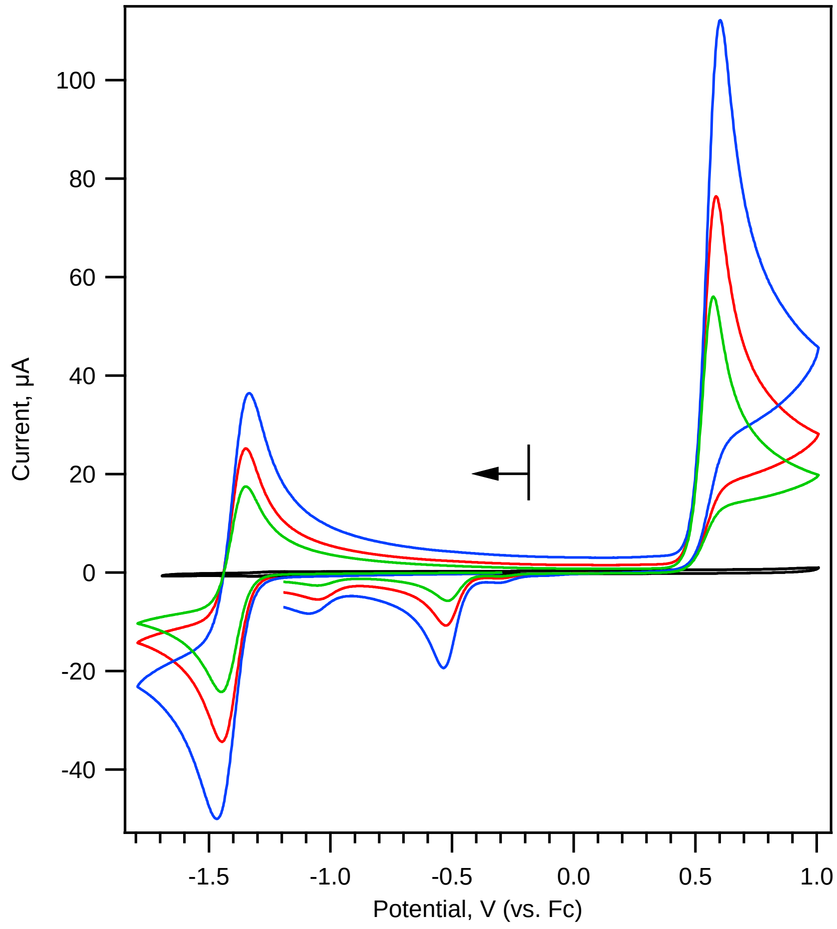

Fig. S2 Cyclic voltammogram of 2 mM $[(C_4H_9)_4N]_3[Fe^{III}(CN)_6]$ and 200 mM tetrabutylammonium hexafluorophosphate in dry acetonitrile. Red line: 100 mV/s scan rate. Green line: 50 mV/s scan rate. Blue line: 250 mV/s scan rate. Black line: Background cyclic voltammogram of 200 mM tetrabutylammonium hexafluorophosphate in dry acetonitrile. 100 mV/s scan rate.

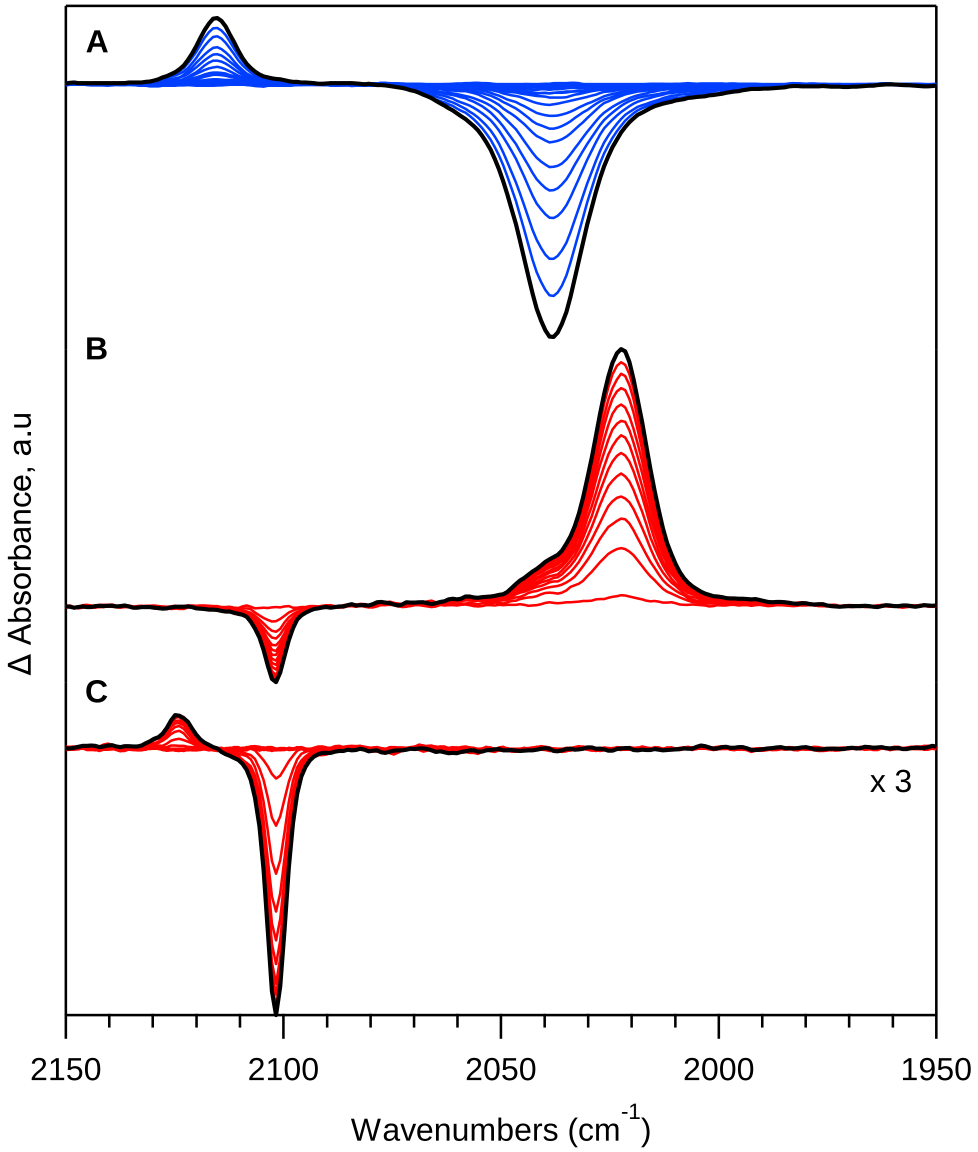

Fig. S3 IR-SEC difference spectra of (A) oxidation of $K_4[Fe^{II}(CN)_6]$ to $K_3[Fe^{III}(CN)_6]$ in water solvent. (B) Reduction of $[(C_4H_9)_4N]_3[Fe^{III}(CN)_6]$ to $[(C_4H_9)_4N]_4[Fe^{II}(CN)_6]$ in dry acetonitrile. (C) Oxidation of $[(C_4H_9)_4N]_3[Fe^{III}(CN)_6]$ to **ox-ferri** in dry acetonitrile. Negative bands denotes loss of starting material while positive bands indicate formation of electrolysis products.

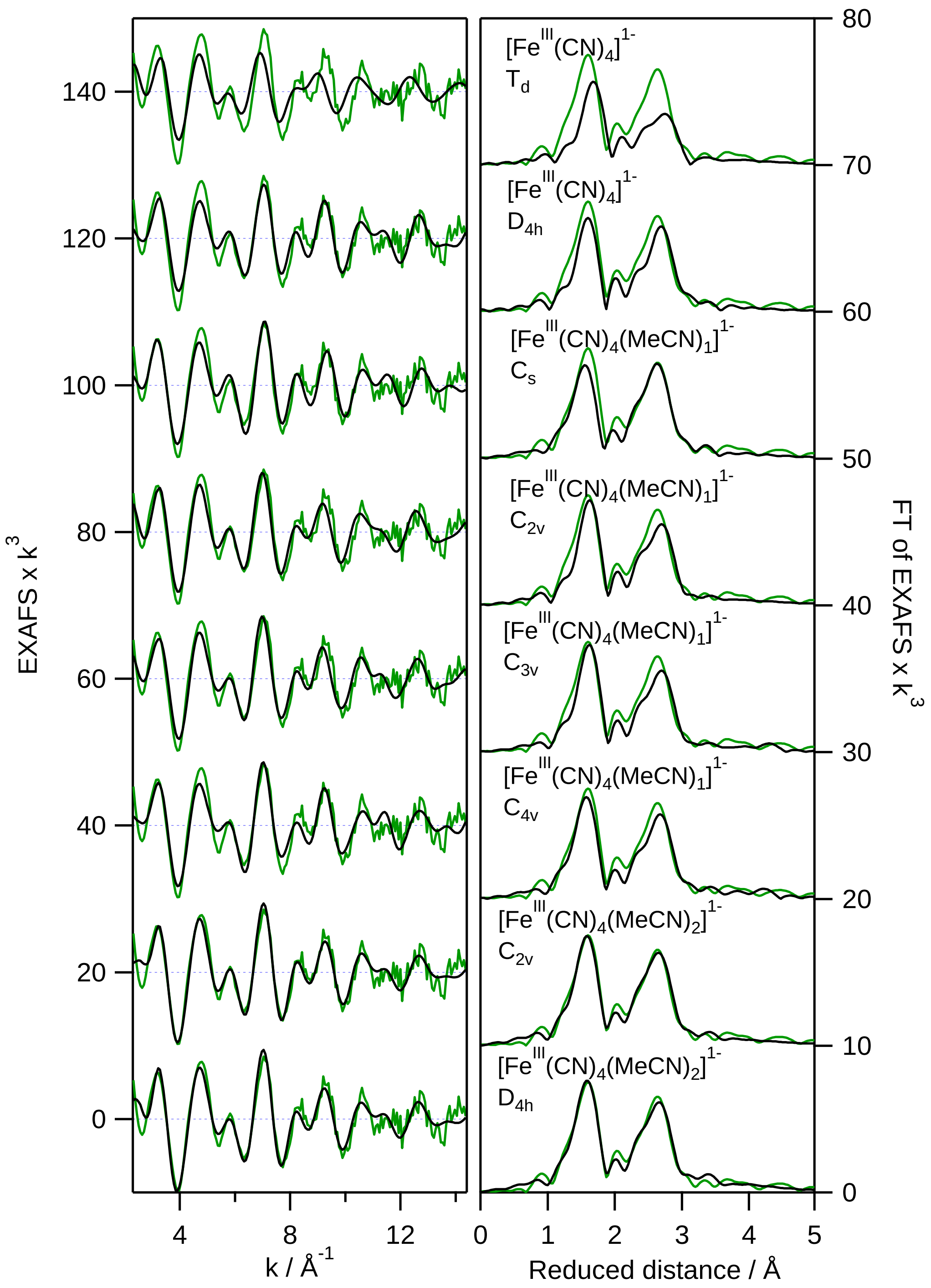

Fig. S4 Experimental EXAFS of **ox-ferri** (green lines) and simulations of EXAFS using DFT optimized structures of $[Fe^{III}(CN)_4(MeCN)_x]^{1-}$, x = 1 or 2 species (black lines). Simulation parameters are given in Table S3. Data offset for clarity.

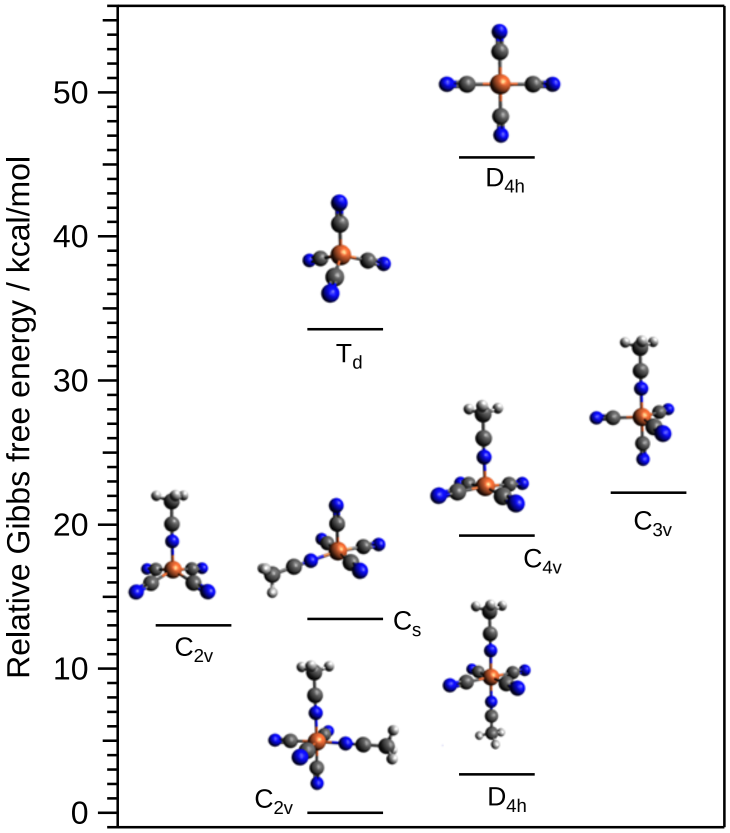

Fig. S5 DFT optimized structures and relative Gibbs free energy of $[Fe^{III}(CN)_4(MeCN)_x]^{1-}$, x = 0, 1 or 2. The isomers are labelled based on their respective idealized symmetry about the Fe center.

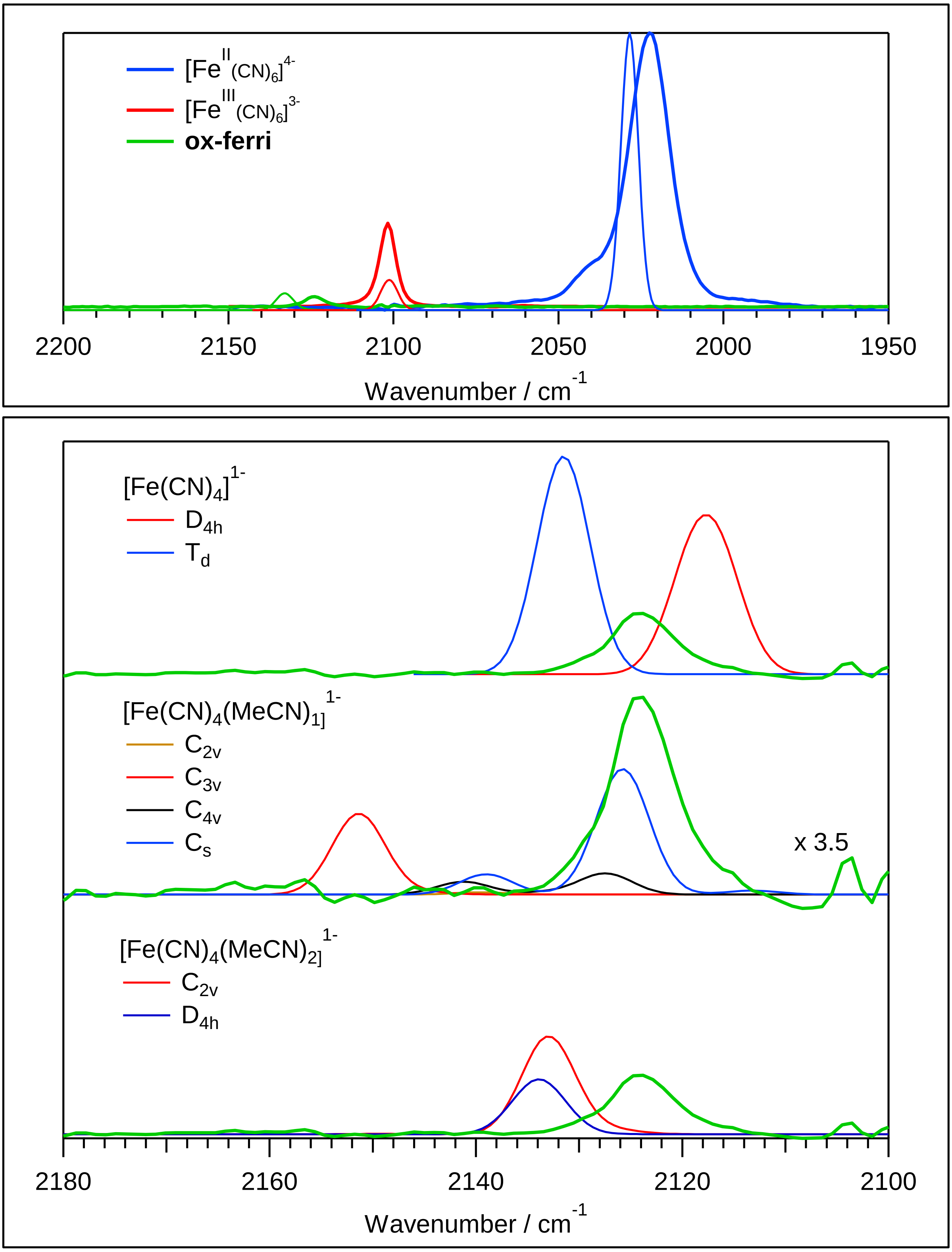

Fig. S6 Top panel, experimental IR spectra obtained from IR-SEC (thick lines) and simulated IR spectra of $[Fe^{II}(CN)_6]^{4-}$ (blue), $[Fe^{III}(CN)_6]^{3-}$ (red), cis-$[Fe^{III}(CN)_4(CH_3CN)_2]^{1-}$ (green) from DFT calculations (thin lines). Bottom panel, simulated IR spectra of various geometry optimized $[Fe^{III}(CN)_4(MeCN)_x]^{1-}$, x = 1 or 2 species (thin lines). Experimental IR spectrum of **ox-ferri** plotted as thick green lines.

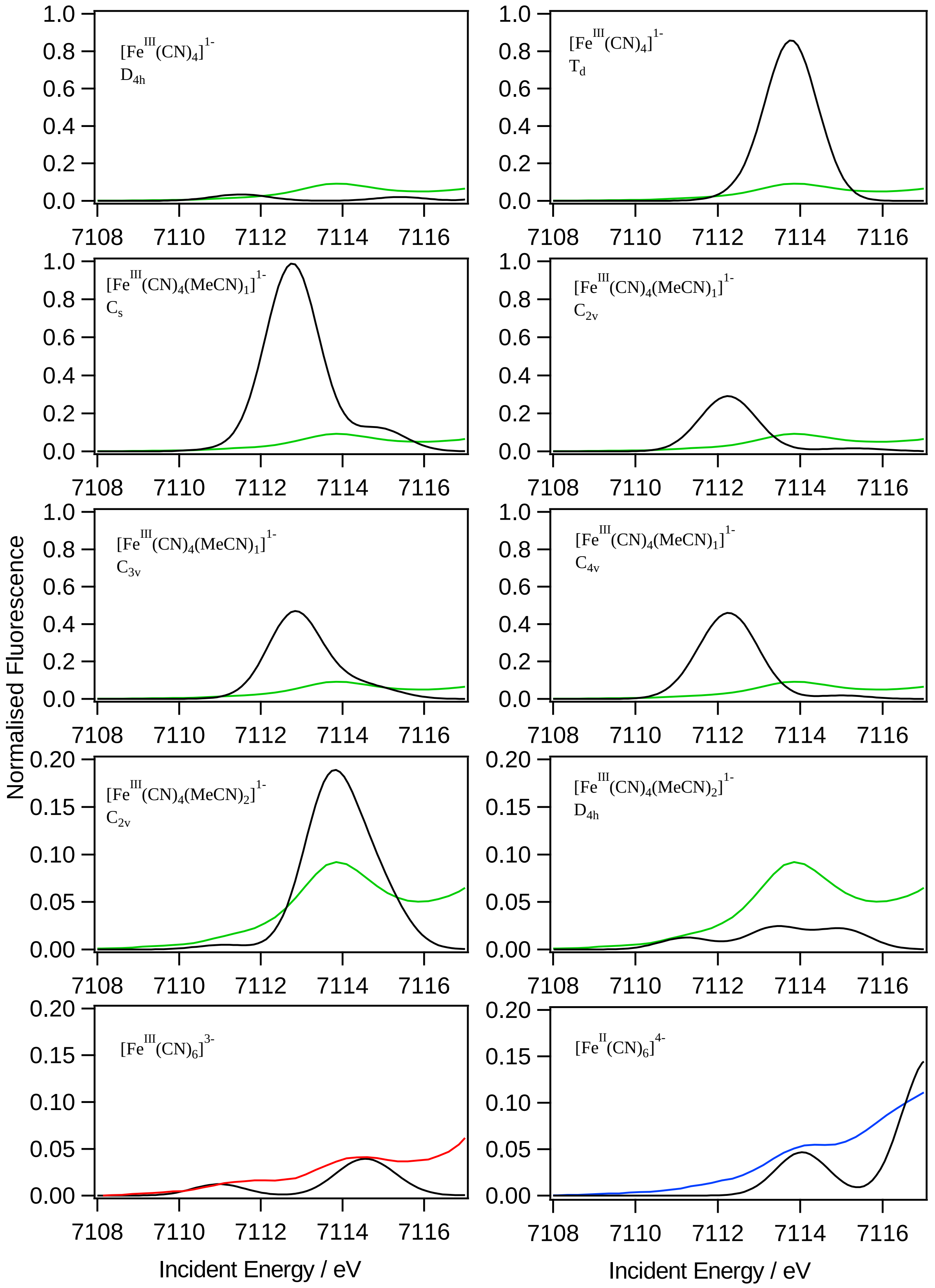

Fig. S7 Fe K-edge 1s → 3d pre-edge region for $[Fe^{II}(CN)_6]^{4-}$ (blue line), $[Fe^{III}(CN)_6]^{3-}$ (red line) and **ox-ferri** (green line). Simulated spectra plotted in black lines.

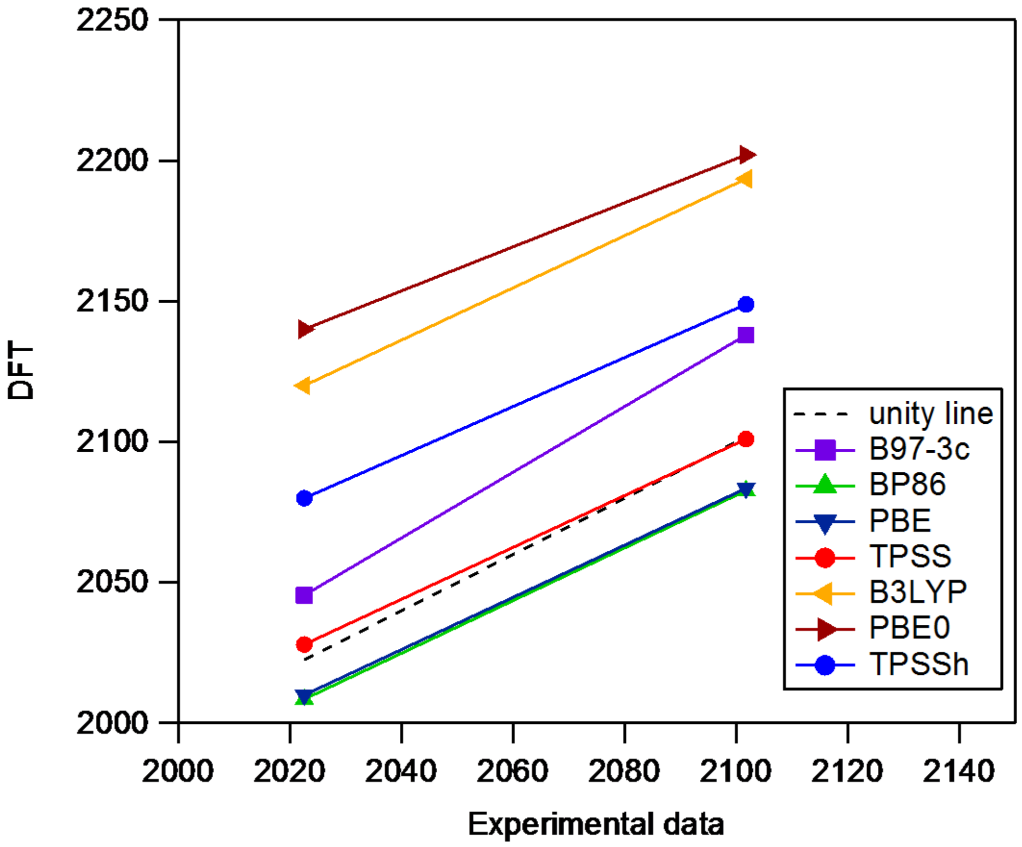

Fig. S8 Comparison between calculated and experimental νCN vibration frequencies for $[Fe^{II}(CN)_6]^{4-}$ and $[Fe^{III}(CN)_6]^{3-}$ with different DFT functionals.

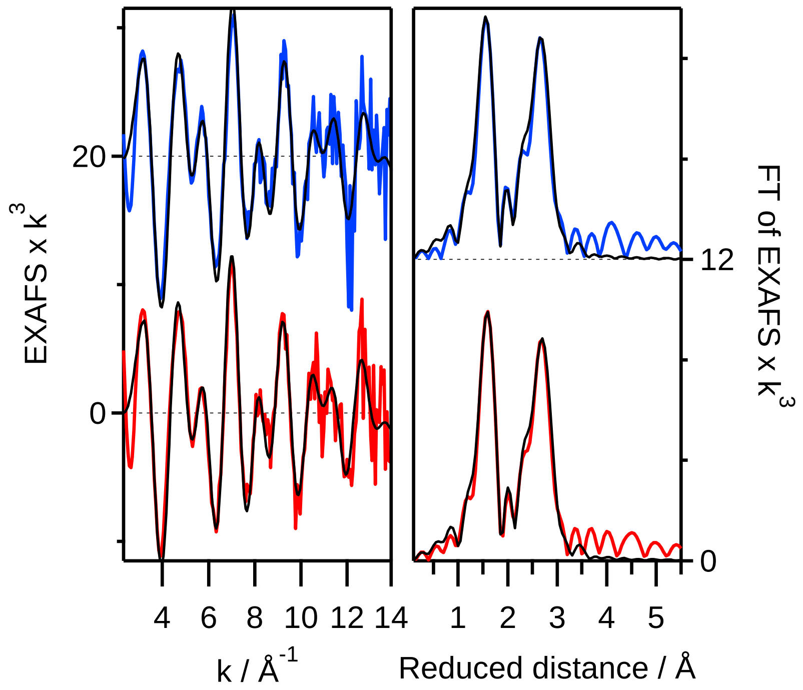

Fig. S9 EXAFS (k^3-weighted) of $[Fe^{II}(CN)_6]^{4-}$ (blue), $[Fe^{III}(CN)_6]^{3-}$ (red). Data offset for clarity. Simulated spectra plotted in black lines. Simulations include two shells (C and N) plus multiple-scattering paths (Fe-C-N and Fe-C-N-C), with fitted distances and Debye-Waller parameters for each shell. Simulation parameters given in Table S2.

	$[Fe^{II}(CN)_6]^{4-}$			$[Fe^{III}(CN)_6]^{3-}$		
	Fe-C	Fe-N	RMSD	Fe-C	Fe-N	RMSD
**EXAFS fitting**	**1.911**	**3.093**		**1.936**	**3.11**	
B97-3c	1.89	3.067	0.019296	1.912	3.081	0.021733
BP86	1.904	3.088	0.004967	1.924	3.1	0.009018
PBE	1.903	3.086	0.006137	1.923	3.099	0.009832
TPSS	1.917	3.097	0.004163	1.933	3.106	0.002887
B3LYP	1.946	3.115	0.023868	1.946	3.108	0.005888
PBE0	1.919	3.088	0.005447	1.922	3.084	0.017049
TPSSh	1.922	3.097	0.006758	1.932	3.1	0.006218

Table S1 Comparison between Fe-C and Fe-N distance obtained from geometry optimized structures of $[Fe^{II}(CN)_6]^{4-}$ and $[Fe^{III}(CN)_6]^{3-}$ using various DFT functionals to distance obtained from EXAFS fitting presented in Table S2.

	$[Fe^{II}(CN)_6]^{4-}$		$[Fe^{III}(CN)_6]^{3-}$	
	6 Fe-C	6 Fe-N + MS	6 Fe-C	6 Fe-N + MS
R / Å	1.911(5)	3.093(6)	1.936(5)	3.110(6)
σ / Å	0.059(4)	0.075(3)	0.055(4)	0.075(3)

Table S2 EXAFS fit parameters for $[Fe^{II}(CN)_6]^{4-}$ and $[Fe^{III}(CN)_6]^{3-}$ used to determine Fe-C and Fe-N distances in the two reference structures. Coordination numbers were kept at 6. R is the interatomic distance, and σ is the Debye-Waller parameter. Multiple-scattering paths included were Fe-C-N (and Fe-N-C, degeneracy 6+6) and Fe-C-N-C (degeneracy 6) and had the same σ as the Fe-N shell. Numbers in parentheses specify the fit error (68 % confidence level) in the last digit. The amplitude reduction factor S_0^2 was fixed at 0.84. Scattering amplitudes and phases were calculated with FEFF 9.0.

	σ first shell / Å	σ other shells / Å	Filtered R-factor / %
$[Fe^{II}(CN)_6]^{4-}$	0.049(5)	0.065(4)	14
$[Fe^{III}(CN)_6]^{3-}$	0.045(5)	0.065(4)	13
$[Fe^{III}(CN)_4]^{1-}$ T_d	0.052(8)	0.100(9)	63
$[Fe^{III}(CN)_4]^{1-}$ D_{4h}	0.045(7)	0.067(5)	37
$[Fe^{III}(CN)_4(MeCN)_1]^{1-}$ C_{2v}	0.045(7)	0.083(6)	36
$[Fe^{III}(CN)_4(MeCN)_1]^{1-}$ C_{3v}	0.043(7)	0.070(6)	35
$[Fe^{III}(CN)_4(MeCN)_1]^{1-}$ C_{4v}	0.054(7)	0.064(6)	34
$[Fe^{III}(CN)_4(MeCN)_1]^{1-}$ C_s	0.050(8)	0.063(5)	34
$[Fe^{III}(CN)_4(MeCN)_2]^{1-}$ C_{2v}	0.060(6)	0.079(5)	20
$[Fe^{III}(CN)_4(MeCN)_2]^{1-}$ D_{4h}	0.067(5)	0.076(5)	23

Table S3 Debye-Waller parameters (σ, in Å) and goodness-of-fit value (R_f, in %) obtained from EXAFS fitting of DFT-optimized models of $[Fe^{II}(CN)_6]^{4-}$, $[Fe^{III}(CN)_6]^{3-}$ and **ox-ferri**. Coordination numbers and distances were kept as in the DFT structures. Scattering amplitudes and phases were calculated with FEFF 9.0. Paths including only atoms from the first shell (at distances around 2 Å) had the same σ; paths that included other shells (at distances around 3 Å and higher) had another σ. Multiple-scattering paths, up to 6 legs, were determined by FEFF 9.0. Numbers in parentheses specify the fit error (68 % confidence level) in the last digit. The amplitude reduction factor S_0^2 was also fitted for the two reference structures ($[Fe^{II}(CN)_6]^{4-}$ and $[Fe^{III}(CN)_6]^{3-}$), and the value obtained, 0.71(3), was then used (not fitted) for the **ox-ferri** models. The filtered R-factor is calculated for the range 0 – 3.5 Å.

xyz coordinates for DFT optimised $[Fe^{II}(CN)_6]^{4-}$, $[Fe^{III}(CN)_6]^{3-}$, cis-$[Fe^{III}(CN)_4(CH_3CN)_2]^{1-}$

$[Fe^{II}(CN)_6]^{4-}$

Fe	-0.00000755204847	0.00000003652302	-0.00014192840861
C	-0.00047311349937	0.00000032281356	1.91641325645959
N	0.00000479016225	0.00000040042321	3.09682230030619
C	-0.00000791161869	-1.91648886046670	-0.00014264344960
N	-0.00000838986848	-3.09690229866253	-0.00014217279015
C	-1.91658288540312	0.00000002218158	-0.00040690094920
N	-3.09701483330866	-0.00000002199940	0.00121450812236
C	0.00048063250240	-0.00000028076007	-1.91671027329896
N	0.00004778515372	-0.00000036187500	-3.09711954021179
C	-0.00000775105477	1.91648896879941	-0.00014344119761
N	-0.00000797707913	3.09690240560840	-0.00014108304789
C	1.91656818816236	0.00000014191530	0.00043613703479
N	3.09699977525551	0.00000041536584	0.00006126678932

$[Fe^{III}(CN)_6]^{3-}$

Fe	0.00001196072325	-0.00000156112217	0.00000749759592
C	0.00006125297980	0.00000535757674	1.93314179873842
N	0.00019444741500	0.00001127076000	3.10582861004711
C	0.00000921008355	-1.93313051429815	0.00001276902166
N	0.00000425190274	-3.10580839560428	0.00001796819951
C	-1.93316484695835	-0.00000310563195	-0.00017444772205
N	-3.10585131400997	-0.00000567084524	-0.00021184373621
C	-0.00007253024729	0.00000069122091	-1.93312599264427
N	-0.00026700474043	0.00000274406009	-3.10581281959120
C	0.00000994480007	1.93312720871359	0.00001290780343
N	0.00000854765343	3.10580511984595	0.00002984685837
C	1.93319010382649	-0.00000171844739	0.00014934698606
N	3.10587654775347	-0.00000178225789	0.00012430289319

cis-$[Fe^{III}(CN)_4(CH_3CN)_2]^{1-}$

Fe	0.01003540405690	0.01640865597817	-0.01557729227314
C	0.02206139697750	1.91349002592943	-0.05492192558886
N	0.02958561778825	3.08350166478118	-0.05608099058948
C	1.95159224981457	0.01615162958813	-0.03143094253855
N	3.12170738012266	0.00952632457339	-0.03590573642393
C	0.00674012055789	0.09044350362142	-1.91168123847361
N	0.00391257349831	0.11357570278498	-3.08148618391325
C	-1.93147623375984	0.04096399921175	-0.02424855026262
N	-3.10157618073484	0.04935513105337	-0.02297868765399
N	0.01159154084110	0.02190379280974	1.93325909504001
C	0.00782116770558	0.02199970025898	3.08721373917844
C	0.00276333329647	0.02008900917402	4.53231871745946
H	0.64062569761428	-0.79223698792559	4.89343144634276
H	-1.01971358123046	-0.12101085070783	4.89405151763541
H	0.39322035432230	0.97711487878814	4.89196026196628
N	-0.00288485802138	-1.93200072378568	-0.05587332224306
C	-0.01212769235051	-3.08571342970904	-0.07809132973256

C	-0.02489465170786	-4.53062355584808	-0.10057262724135
H	-0.01472174319074	-4.90185140086984	0.92901862311998
H	-0.93062196615408	-4.87849411785691	-0.60576649588886
H	0.86063207055387	-4.89475257684964	-0.62954632791919
